# The prevalence and clinical presentation of antenatal depression in rural South Africa

**DOI:** 10.1016/j.jad.2011.08.011

**Published:** 2011-12

**Authors:** Tamsen Jean Rochat, Mark Tomlinson, Till Bärnighausen, Marie-Louise Newell, Alan Stein

**Affiliations:** a Africa Centre for Health and Population Studies, University of KwaZulu-Natal, South Africa; b Department of Psychology, Stellenbosch University, South Africa; c Department of Global Health and Population, Harvard School of Public Health, Boston, United States; d MRC Centre of Epidemiology for Child Health, UCL Institute of Child Health, London, United Kingdom; e Section of Child and Adolescent Psychiatry, Department of Psychiatry, Oxford University, Oxford, United Kingdom

**Keywords:** Depression, Antenatal, Postnatal, Pregnancy, HIV, South Africa

## Abstract

**Background:**

Although the prevalence of depression is similar in pregnant, postpartum and non-pregnant women, the onset of new depression is higher during the perinatal period. Women of low-income, and those living in low and middle income countries, are known to be at particularly high risk. Early identification and treatment of antenatal depression may improve pregnancy outcomes and could serve as an early indicator of postnatal depression. Culturally sensitive and accurate diagnostic tools are urgently needed.

**Methods:**

A consecutive series of 109 pregnant women were recruited in the third trimester at a primary health clinic, in a rural part of South Africa, with a high HIV prevalence. A cross sectional assessment of depression was completed using a structured clinical interview method and DSM-IV diagnostic criteria. Qualitative data on women's descriptions of depressive symptoms was also collected. The aim was to examine the prevalence of depression and to better understand the presentation of depressive symptomatology in this population.

**Results:**

Prevalence of depression was high, 51/109 (47%), with over half of the depressed women 34/51(67%) reporting episode duration greater than two months. 8/51 reported a prior history of depression. Women used psychological language to describe symptoms and, as a result, standardised diagnostic tools were culturally sensitive. Somatic pregnancy symptoms were frequently reported, but did not overestimate depression. Both HIV positive (27/51) and HIV negative (24/51) women were at risk of being depressed.

**Limitations:**

The study is limited by the small sample size and possible attrition biases.

**Conclusion:**

Antenatal depression is high and clinical presentation is similar to high income countries. Standardised diagnostic tools are culturally sensitive and adequate for early detection.

## 1 Introduction

The onset of new depression is higher during the perinatal period and is of growing concern because of its impact on both mother and child. Community based samples show that women in the antenatal period are not significantly more vulnerable to depression than their non-pregnant or postnatal counterparts (Vesga-Lopez et al., 2008), however, women of low-income and those living in low and middle income countries are known to be at particularly high risk of both antenatal and postnatal depression (Bennett et al., 2004). Depression during the perinatal period is associated with a range of foetal and obstetric problems and adverse developmental child outcomes (Alder et al., 2007), and in low and middle income countries evidence suggests that its impact extends beyond psycho-social developmental delays to child health outcomes (World Health Organisation, 2009). Despite these known risks, depression during the antenatal period is often not recognised and treatment rates are lower in pregnant than in non-pregnant women; even in well resourced contexts (Vesga-Lopez et al., 2008). Early identification and treatment of antenatal depression may improve pregnancy outcomes and could help to prevent postnatal depression.

A growing body of literature suggests that antenatal depression is at least as common as postnatal depression and meta-analyses have shown that antenatal depressive symptomology is a strong predictor of postnatal depression (Lancaster et al., 2010). In a community-based sample of 14,000 women in the United Kingdom most postnatal depression was preceded by antenatal depression or anxiety and, when antenatal symptoms were present, a higher number of symptoms were reported relative to the postnatal period (Evans et al., 2001; Heron et al., 2004). In a large prospective cohort of women in Australia antenatal symptoms were as common as postnatal symptoms. Depression during pregnancy, or a previous history of depression outside of pregnancy, were significant predictors of postnatal depression (Milgrom et al., 2008). Among Chinese women and rural Pakistani women, depressive symptoms in the second and third trimester had a strong relationship to postnatal depression 6 weeks postnatally (Lau et al., 2010) (Rahman and Creed, 2007). While significant evidence exists on the onset, course and duration of postnatal depression, less is known about antenatal depression. The few studies available point to an increased vulnerability to depression in the latter part of pregnancy. A recent meta-analysis found that the rate of depression in the first trimester was similar to rates seen in the general female population, while rates in the second and third trimester were double those observed in the general population (Bennett et al., 2004). This pattern of third trimester vulnerability has also been reported in studies from Asia and Africa and appears to be particularly heightened in low income settings where prevalence's between 35 and 50% have been observed in the third trimester (Abiodun, 2006; Esimai et al., 2008; Lau et al., 2010). A systematic review of pre- and postnatal psychological well-being in Africa, found only 13/35 studies from five countries examined psychological disorders during pregnancy, pointing to the paucity of research on the antenatal period in Africa and in sub-Saharan Africa in particular (Sawyer et al., 2010). No studies in South Africa have examined antenatal depression, but those examining postnatal depression have found rates between 34 and 48% (Cooper et al., 2009; Madu and Roos, 2006). The high burden of postnatal depression in South Africa likely relates to women's exposure to multiple depression-related risk factors, such as poverty, intimate partner violence and the increasing threat of HIV (Brandt, 2009; Dunkle et al., 2004; Jewkes et al., 2010; Stein et al., 2005).

While there is a clear need for further research, it has been suggested that the use of standardised western methods and diagnostic systems in non-western contexts may be culturally insensitive and could increase the risk of over- or under-reporting depression in these contexts (Halbreich et al., 2007; Halbreich and Karkun, 2006). There is now general consensus that psychological symptoms can be easily elicited (Patel et al., 2001) and it is suggested that an overemphasis on possible cross-cultural differences may undermine the allocation of resources for treatment in settings where it is most needed. An expanding literature has demonstrated that postnatal depression occurs in a variety of countries, including those with traditional cultures (Posmontier and Horowitz, 2004). However, culturally sensitive and accurate diagnostic tools are urgently needed (Patel et al., 2009; Prince, 2008).

This study aimed to determine the prevalence of antenatal depression using a gold standard clinical interview method. We aimed to identify which symptoms were most frequently reported amongst both depressed and non-depressed women, and used qualitative methods to provide a clear and culturally sensitive understanding of the clinical presentation of depression in pregnant women in a rural area of South Africa.

## 2 Method

### 2.1 Research context

The research was undertaken in a predominantly rural area of South Africa with high HIV prevalence (Barnighausen et al., 2008; Tanser et al., 2008). The Hlabisa sub-district health services include a local district hospital with 296 beds and 17 decentralised primary health care clinics servicing a population of approximately 220,000 (Houlihan et al., 2011). This study was undertaken at the largest primary health care clinic in the sub-district. The clinic was staffed by 20–30 nurses and offered the full range of primary health care services to over 10,000 patients per month. This includes a large antenatal and postnatal outpatient service with an average of 160 first time antenatal attendees per month. It was the only clinic in the sub-district offering a 24 hour service, with between 70 and 100 deliveries per month.

### 2.2 Recruitment procedure

**Fig. 1:**
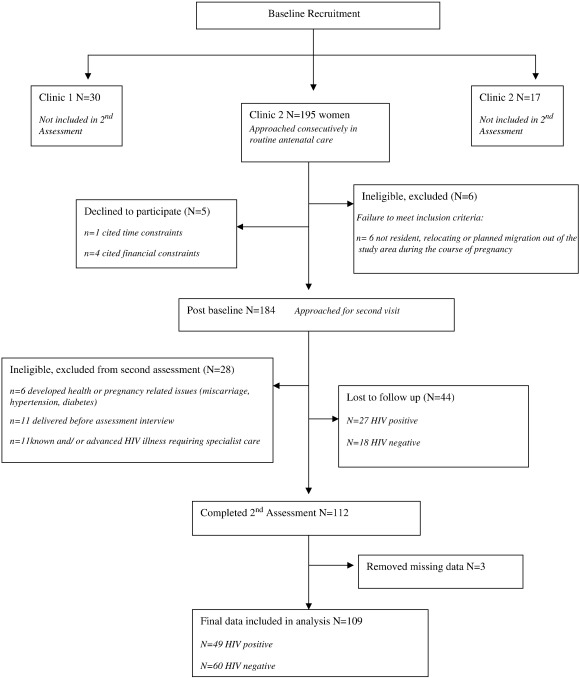
Baseline Recruitment.

This nested study recruited a sub-sample of women from a larger cohort of baseline participants enrolled at three clinics in the sub-district (Rochat et al., 2006) as outlined in Fig. 1, and data collection was completed in 2006. Women were invited to participate in an in-depth assessment interview 3 to 4 weeks after baseline. The research was cross-sectional and descriptive. To be eligible, women were required to be pregnant, attending routine antenatal care, be at least 16 years of age, in the second half of pregnancy, and live in the study area. Women with severe or chronic health problems such as diabetes or a known HIV-related illness were referred out of the primary health care setting for specialist services and excluded from this research. Informed consent was obtained in writing and the study received ethical approval from the Biomedical Ethics Review Board of the University of KwaZulu-Natal and the Oxford Tropical Research Ethics Committee (OXTREC).

### 2.3 Depression assessment

Two South African IsiZulu-speaking researchers were trained to interview mothers using the major depression section of the structured clinical interview for DSM-IV diagnoses (American Psychiatric Association, 2000). This assessment method has been used previously in a clinical trial of postnatal depression in South Africa (Cooper et al., 2009). The interview questions were translated into IsiZulu and back translated. To ensure the accuracy (sensitivity and specificity) of symptom descriptions in the local language each of the DSM-IV symptoms was extensively explored in a workshop with both English and IsiZulu-speaking clinical psychologists and psychiatric nurses. During the interview, extensive qualitative data were recorded in the form of interview notes using the ‘*what has this been like for you*’ item to explore cultural aspects which might inform the measurement and understanding of antenatal depression. This mixed methods approach, using both quantitative and qualitative data collection techniques is considered important in developing our understanding of health and health behaviours (Deren et al., 2003). Quantitative methods are able to measure the duration, number, and presence or absence of symptoms according to a standardised definition of depression. However, these methods might not be able to capture the contextual factors that affect the experience of depression or the meaning that depression has to the pregnant women. Qualitative studies about depression can describe the experience of depression from the point of view of the person, but they cannot describe the distribution, magnitude, or frequency of that experience at a group or population level. Mixed methods approaches provide an approach by which researchers can attempt to understand the experience of depression among pregnant women from different and complementary conceptual frameworks, as has been demonstrated in research on the experience of depression among the elderly (Barg et al., 2006). To monitor the quality of these notes, 56 (50%) of the 112 interviews were tape recorded, transcribed and translated. The first author (a clinical psychologist) reviewed each assessment using the tape recordings (where available), item responses and the detailed interview notes to code and score symptoms. Under supervision by the last author (a psychiatrist) decisions were made about the presence or absence of a DSM-IV major depressive disorder.

### 2.4 Data analysis

A major depressive episode (MDE) was defined in line with DSM-IV diagnostic guidelines. The interview data was coded by symptom and reduced to a dichotomous 0/1 score based on an evaluation of the individual symptoms presence, severity and duration. An overall depression (0/1) outcome was then determined using the DSM diagnostic criteria.

#### 2.4.1 Quantitative analysis

Data was double entered for accuracy into STATA10 software for data analysis. Exploratory factor analysis examined whether the various symptoms of depression reflected a single or multiple underlying latent variable(s) of depression. Data was entered and analysed in its most disaggregated form with each of the individual symptoms and its sub-classifications entered separately. For example, sleep disturbance (which can include delayed sleep, middle insomnia, early waking and hypersomnia) was entered with all four sub-classifications. Principal component analysis techniques were used to examine groups or clusters of symptom variables around a main or principal component, and to examine the spread of symptom variance within that component.

#### 2.4.2 Qualitative analysis

The qualitative data analysis used content analysis to identify categories of responses for each depressive symptom. Once common categories were identified and coded these were assessed using a constant comparative analysis technique to determine summary concepts within each category which were inclusive and mutually exclusive. Categories of coded data were entered, organised and analysed with NVivo software to establish counts and frequencies illustrating common language, terminology and conceptual representations of depression symptoms.

## 3 Results

### 3.1 Sample characteristics

One hundred and ninety five antenatal attendees enrolled in a baseline screening study (Rochat et al., 2006) were approached to complete a second in-depth assessment (see Fig. 1). Of these, five women refused to participate citing time or financial constraints and six women did not meet the eligibility criteria. Of 184 eligible women, a further 17 were excluded post baseline enrolment due to pregnancy complications including preeclampsia, diabetes, advanced HIV illness or miscarriage. A further 11 women were unable to participate as they delivered their babies before the second assessment. Of the remaining 156 eligible women, 112 (72%) completed the assessment and 44 (28%) were lost to follow up. Although a third more HIV positive women were lost to follow up than HIV negative women, an examination of data available from the baseline showed no significant distinguishing characteristics among women lost to follow up as compared to those interviewed. A further three women were removed during data analysis as a result of missing data, leaving a final sample of 109 women included.

The characteristics of the 109 women are outlined in Table 1. Most women were young (aged 16 to 28 years), had completed some or all of their secondary education, and were from low-income groups. They were primarily unmarried but in a stable relationship with the father of the child, and most were living with their families rather than co-habiting with partners. While a few participants had enrolled at 26 weeks, most participants had enrolled between 28 and 34 weeks gestation. 85% of women had an unplanned pregnancy. Nearly half (49/109 or 45%) of the women were HIV positive; in line with what was expected given the high antenatal HIV prevalence in this health district (Rice et al., 2007; Department of Health, 2010).

### 3.2 Prevalence of depression

High numbers 51/109 (47% CI 37.2–56.3) of women met the criteria for a major depressive episode (MDE). Of these, 14/51 (28%) reported episodes lasting between two weeks and two months, while 34/51 (67%) reported the current episode had persisted for more than two months but less than 6 months. Eight of the 51 (16%) depressed women reported a previously diagnosed episode of MDE which resolved prior to the current pregnancy, two of which were reported to have occurred during the postnatal period of a previous pregnancy. Univariately, there was no significant relationship between previous episodes of depression and depression outcome (OR 1.35 CI 0.4–4.0 p = 0.585), although the numbers were too small for this to be conclusively examined in data analysis.

### 3.3 Frequency of depressive symptomology

**Fig. 2:**
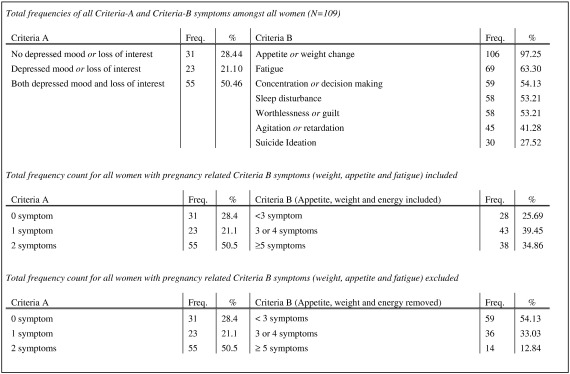
Frequency of criteria-A and criteria-B symptoms of depression.

Fig. 2 summarises symptom frequencies for the presence of criteria-A and criteria-B symptoms for women, both with and without depression. The reporting of symptomatology was generally high with the mean number of symptoms 5.11 (SE 0.23 CI 4.64–5.59). Frequencies of criteria-A and criteria-B symptomatology are listed from highest to lowest frequency. Weight, appetite and fatigue were the most frequently reported symptoms. Since these symptoms are frequently confused with normal pregnancy related physiological changes and can overestimate depressive symptoms, symptom frequencies were analysed with and without these symptoms, as shown in Fig. 2. When these symptoms are excluded 46% of women still scored positive for the presence of at least 3 or more criteria-B symptoms.

### 3.4 Clinical presentation of depression

**Fig. 3:**
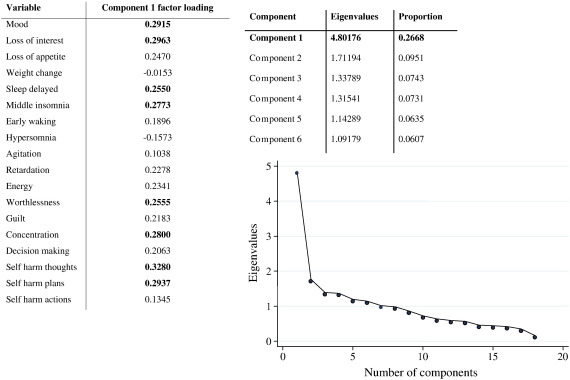
Scree plot with principle component, eigenvalues and factor loadings.

Principal component analysis extracted one principal component based on scree plot inspection, see Fig. 3. This component had factor loadings falling closely together on a continuum between 0.15 and 0.35 across all the variables in the component, suggesting that in the underlying structure of the data all of the symptoms included in the DSM-IV assessment of depression (with the exception of weight change) are likely relevant to the clinical presentation of depression. Factor loadings cluster into three groups (< 0.1; 0.1–0.2 and > 0.2) within the principal component, although it is noteworthy that the difference between these clusters is small. The principal component has eight variables with higher factor loadings (> 0.25) representing six depressive symptoms (mood, loss of interest, sleep disturbance, worthlessness, concentration and suicide ideation) which are described in greater detail in this paper. Suicidal ideation has the highest factor loading, followed closely by mood and loss of interest, and then by a cluster of sleep disturbance, concentration and worthlessness.

### 3.5 Qualitative descriptions of depression symptoms

#### 3.5.1 Depressed mood and loss of interest

As shown in Fig. 2 reporting of depressed mood and loss of interest tended to overlap, with the majority of depressed women reporting both criteria-A symptoms. Table 2 shows that over half (68/109) of the women in the sample reported depressed mood and 49/68 of those were diagnosed with MDE. Similarly 65/109 reported loss of interest, 45/65 of who were subsequently diagnosed with MDE. Disturbances of mood (0.2915) and loss of interest (0.2963) were two of the higher factor loadings in the principal component.

**Fig. 4:**
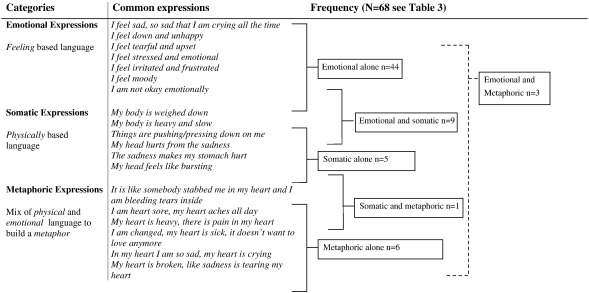
Categories used to describe depressed mood.

Content analysis of interview notes showed that women used one of three categories to describe their experiences of depressed mood, see Fig. 4. Most women (44/68 or 65%) reporting depressed mood did so using psychological constructs and language:

*“I am not okay, I mean physically I am okay, but emotionally I am not okay” (P79)*

The most frequently used word to describe depressed mood was feeling of ‘sadness’ reported by 39/44 (89%) of the women using emotional expressions to describe depressed mood. In examining the concept of sadness more closely, results show that among the 39/44 women reporting sadness, 36/39 (92.3%) had made reference to excessive crying or tearfulness as a means to describe the depth or nature of their feeling of sadness:

*“I feel so sad and I end up crying, and at times I cry without any sound reason” (P91)*

Sadness was a pervasive concept which emerged in all three categories and was seldom reported in isolation. A small group of women (16.0%) reported feeling low or down, making direct reference to the construct of depressed mood as it is described in the DSM-IV:

*“My mood is getting lower and lower, and I feel very down and I don't enjoy things” (P38)*

As compared to emotional expressions alone, only 6/68 women (8.8%) described being depressed using metaphorical descriptions alone and 5/68 women (7.3%) described depressed mood using only somatic expressions. Small numbers of women reported depressed mood using both emotional and metaphoric concepts (3/68) or somatic and metaphoric (1/68). Instead women who combined emotional expressions with other conceptual categories were more likely to combine emotional with somatic expressions 9/68 (13.2%), such as describing sadness together with headaches and bodily aches and heaviness:

“*I feel very sad all the time, like I am weighed down, like my body is heavy and I don't feel like talking” (P83)*

Content analysis of responses to the loss of interest item (*“What kinds of things have you lost interest or pleasure in?”*) showed that women most commonly reported loss of interest or pleasure in relation to three activities: spending time in the company of family and friends; their relationships with their partners both emotionally and sexually; and taking part in specific social activities such as attending church functions or celebratory events such as weddings and birthday parties.

#### 3.5.2 Suicide ideation

High numbers of women 30/109 (28%) reported suicide ideation, most of whom (27/30) were also depressed. Women reporting suicide ideation tended to express this as ‘*I would be better off dead*’ rather than directly stating they intended ‘*to commit suicide or harm themselves*’. Among suicidal women, most (24/30) had both and planned how they might harm themselves while 2/30 women reported previous suicidal attempts. Suicidal thoughts were also the highest criteria-B factor loading (0.3280) followed closely by suicidal plans (0.2937).

Suicide ideation was shown to reflect a strong sense of hopelessness. Commonly reported plans towards self harm included: drinking paraffin or poison; drowning; hanging; burning; or throwing oneself in front of a car.

*“I feel like disappearing and leaving everything behind, that I should be dead so it can all go away” (P40)*

*“It would be better if I wasn't around because I can't deal with this” (P105)*

*“It's better to end my life things are so bad” (P82)*

#### 3.5.3 Sleep disturbance

58/109 women reported sleep disturbances and more than half of those (38/58) were also diagnosed with MDE. Sleep disturbance, in particular delayed sleep (0.2550) and middle insomnia (0.2773) had high factor loadings.

Results of content analysis of SCID responses on sleep disturbance for the item *“what has caused you difficulty in sleeping”* found that depressed women were most likely to report that thoughts and worries were interfering with their ability to sleep while non-depressed women tended to report pregnancy related reasons for sleep disturbance:

*“I find myself thinking and worry about things and then I cannot sleep” (P20)*

*“I am just uncomfortable now, so it happens that I wake up for no reason” (P10)*

#### 3.5.4 Concentration

Fewer women reported concentration difficulties (47/109) relative to suicide and sleep disturbances. However, concentration had a high factor loading (0.2800) and depressed women reported double the amount of concentration (35/51) and decision-making symptoms (28/51) than reported by non-depressed women (12/58 and 14/58 respectively). Content analysis found that women most frequently described their concentration difficulties as feeling ‘easily distracted’ or being ‘forgetful’:

*“In the middle of things, I just forget what I am doing” (P105)*

Since almost all the women were unemployed, difficulties with concentration were often expressed in reference to household tasks, as this was the main daily activity they engaged in:

*“Yes, like shifting backwards and forwards in household tasks and not finishing things” (P30)*

*“Like when I am cooking, I burn my hand without even noticing it” (P82)*

While non-depressed women also related difficulties with concentration, depressed women were distinctive in their descriptions of the severity of difficulties with concentration.

*“I can't manage to do even normal things, like household things, the backbone of my life is gone” (P73)*

*“It's not that bad that my plans get affected, it's just hard to think sometimes” (P64)*

#### 3.5.5 Worthlessness

Over a third (43/109) of women reported feeling worthless; 31/43 (72%) of whom were also depressed. Worthlessness had a high factor loading (0.2555) and women used common western terms to describe their sense of worthlessness. For example, women made statements such as:

*“I feel worthless, it's like I don't have any value and I am not important to anybody” (P105)*

Most women who scored positive for worthlessness cited that a feeling of worthlessness was a recent experience, representing a significant change or shift in how they felt about or perceived themselves in recent weeks and months. Women frequently reported this change using the construct of pride, self-worth, and self-esteem.

*“Things have changed and I don't feel the same anymore, I don't feel proud of myself” (P27)*

*“I don't feel the same anymore, I feel ashamed of myself” (P38)*

## 4 Discussion

Antenatal depression prevalence was high, similar to that found in a baseline study using the EPDS screening tool in the same community (Rochat et al., 2006). This prevalence is four times higher than the estimated prevalence of 7 to 12% in high income countries (Bennett et al., 2004) and twice as high as estimated prevalence of 4 to 17% in Africa (Sawyer et al., 2010). This estimate is comparable to established risk in the postnatal period in South Africa and similar to rates of 37% found in clinical research among non-pregnant groups in primary health care settings in South Africa (Carey et al., 2003). This research supports the growing body of literature showing that women living in low and middle income countries have a much higher risk of antenatal depression than their high income counterparts.

These findings highlight the importance of routine antenatal screening for depression in primary health care given that antenatal depression is already known to negatively impact on the uptake of antenatal care as well as on foetal and obstetrics outcomes, and is a strong predictor of postnatal depression, (Austin, 2004; Austin and Lumley, 2003). Detection of early symptoms of depression may prevent antenatal depression and may protect against the onset of depression in the postnatal period. However, in lower income settings, where women appear to be at high risk, the practical challenges involved in routine screening, given that resources are often scarce, should not be underestimated (Patel et al., 2007, 2009). Shorter, time efficient and accurate screening methods are important to facilitate universal screening within antenatal care (Smith et al., 2010). Given episode duration and pregnancy gestation, it is likely to be most useful to screen early in the pregnancy and, where possible, to repeat screening, in particular during the course of the second trimester.

Cultural sensitivity in the assessment of depression is important for two reasons. Firstly, contextual factors may influence how individuals in different cultures perceive psychological and social stressors and, secondly, culture and language may influence how antenatal depression is reported, experienced or responded to (Bernazzani et al., 2004; Bina, 2008). Any discussion on cross-cultural validity of diagnostic tools however needs to ensure a balance between sensitivity and validity. If too much local cultural diversity is incorporated into an established diagnostic instrument, the degree of alteration may render the instrument incapable of measuring the original constructs for which it was designed (Canino and Alegría, 2008). In this research, we found little evidence to support a culturally distinct presentation of depression. Women used psychological language in their description of their experience of depression and depressed women were able to make the subtle distinctions between metaphorical and cultural specific descriptions and their depressive symptoms. A recent study in of women's distress during pregnancy in Tanzania reported similar findings, illustrating that while local idioms are important and feature strongly in women's descriptions of depression and distress during pregnancy, the construct of depression in these contexts is similar to bio-medical criteria for depression in other contexts (Kaaya et al., 2010). This is important, as the development of culture-specific assessment methods would be time consuming and expensive (Halbreich and Karkun, 2006). However, cultural aspects may provide particular meaning around beliefs about the cause of a symptom or beliefs about how those symptoms could or should be alleviated or treated. So while they did not strongly influence the assessment of symptoms against DSM-IV criteria, it may be important to consider their influence on the acceptability of particular treatment approaches (Oates et al., 2004). For example how a woman understands or assigns meaning to the cause of depression may influence what she considers to be an appropriate response to depression. This may be a relational or social intervention in response to the cause being seen as related to interpersonal difficulties, a psychological intervention in response to a intrapersonal issue, or a biomedical in response to biochemical imbalances, or indeed a cultural responses (such as a ritual or prayer) in response to an culturally defined issue such as bewitchment or dissatisfied ancestors.

Results of the exploratory principle component analysis show that the clinical presentation of depression in this population likely features the following symptoms: disturbance of mood, loss of interest, suicide ideation and to a lesser extent concentration difficulties, sleep disturbance (specifically delayed sleep and middle insomnia) and worthlessness. The reporting of symptoms which are commonly reported as normal side effects of pregnancy (such as weight gain) did not appear to overestimate the level of depression in this sample, similar to findings from research in Europe (Kammerer et al., 2009). Pregnancy itself did not seem to overly complicate the diagnosis of depression. Women frequently described depression in relation to its effect on their emotions, their interactions with others, and their day to day homemaking functioning. While no significant relationship was found between previous episodes of depression and current depression, the numbers were small and further research with larger community- based samples in low and middle income countries is needed. Research in high income countries shows that pregnancy itself does not hold specific elevated risk for depression. However, there is growing evidence that a previous history of depression significantly elevates risks for depression during the course of pregnancy (Gavin et al., 2005; Lusskin et al., 2007). It is not clear how much of this risk is accounted for by a predisposition alone, or by the biological changes or psycho-social stressors associated with pregnancy and their interactions with existing vulnerabilities, or how much may relate to the cessation of pharmacological treatment in order to facilitate pregnancy (Cohen et al., 2006).It is also unclear whether these risks would apply in low and middle income countries. In this research, it is possible, given historical limitations in the availability of mental health screening and treatment resources, that some women may have had previous episodes of depression which went undetected and untreated, and may thus not have been reported.

In this study, most cases of depression were severe and chronic, suggesting significant disability and lowered functionality. Research among low income African–American women with antenatal depression has shown that depression during pregnancy was strongly associated with a global reduction in functional status and perceived wellbeing (McKee et al., 2001). In that research increasing the number of social supports did not necessarily reduce the impact of depression among severely depressed women, suggesting that for women with severe depression, psychological rather than social interventions may be more appropriate. Severe depression may also impact on health-related functional status, in particular in the context of HIV. Since many studies have suggested untreated depression is associated with lowered uptake (Cook et al., 2006) and lowered adherence to HAART (DiMatteo et al., 2000; Starace et al., 2002); and increased disease progression (Ickovics et al., 2001) there is an urgent need to integrate and include mental health screening and services in both antenatal and prevention of mother to child transmission programmes in primary health care contexts in areas of high HIV prevalence. In areas such as sub-Saharan Africa where HIV prevalence is high, pharmacological interactions between anti-depressants and highly active antiretroviral treatment (HAART) which may increase depressive symptoms also need to be considered, along with possibilities of reduced adherence commonly associated with depression in non-pregnant women. While guidelines for the safe use of pharmacological treatments during pregnancy are now available (Kuehn, 2010) the availability of medication and skilled professionals to monitor pharmacological treatment during pregnancy are scarce or absent in most resourced-limited settings. In this research women with HIV related illness, or those who had become pregnant knowing they were HIV positive or on HAART, were excluded as they were considered to be a separate group with different needs and risk profiles, and because they would not receive routine antenatal care in a primary health care context as the pregnancy progressed but would instead be referred to specialist services. As a result, all the women in this sample were testing for HIV for the first time in the pregnancy as part of routine antenatal care, and large numbers of women (49/109) tested HIV positive. It may be expected that learning one is HIV positive may trigger or increase depression symptomology, however all women had tested for HIV recently (approximately 2 weeks prior and no more than 4 weeks prior) to the depression assessment. Over two thirds of depressed women reported that their depression was chronic in nature and greater than two months in duration. Thus, it is not likely that testing HIV positive alone resulted in high depression rates.

Importantly, the mean level of depressive symptomology reported was high regardless of depression outcome. This pattern of generalised distress among pregnant and postnatal women is not uncommon in low and middle income countries (Rahman et al., 2008b). Increasing evidence suggests that clinical depression may not be significantly distinct from depressive symptomology in terms of its impact on functioning. It has been shown that many measures of psychosocial dysfunction did not significantly differ between false positives and true positives on depression screening measures, suggesting that women with a high number of acute symptoms on a screening tool may be presenting with as much functional risk as women diagnosed with major depression (Gotlib et al., 1995). The elevated levels of reported depressive symptoms in this research dispel concerns, often raised in high income settings, that depression during pregnancy may be highly stigmatised and difficult to detect (Lusskin et al., 2007). However, this raises a different challenge around how to efficiently identify the highest risk groups in resource limited settings, given that treatment of false positives is costly and time consuming. While all women may benefit from treatment, not all women presenting with depressive symptomology may warrant treatment when resources are limited. The costs of unwarranted treatment have been shown to be high and have raised questions around universal screening, even in well resourced settings (Paulden et al., 2009).

These factors, along with other commonly cited barriers to treatment in poorly resourced settings (such as a lack of transport resources to attend care or long waiting periods in public sector care facilities) may all play a large role in reducing the amount depression which is currently detected during pregnancy and in reducing women access to treatment for depression (Kopelman et al., 2008). A lack of health care provider awareness and training has also been shown to account for much inequity in access to treatment for depression (Almond and Lathlean, 2011). Similarly it is probable that depression itself may inhibit a women's engagement with health services during pregnancy. While detection is complicated by these factors, this research indicates that the rate of antenatal depression is high; suggesting that improved detection could hold significant opportunities to effectively treat antenatal depression and to prevent postnatal depression.

## 5 Conclusion

Training, awareness and resources are necessary to ensure that primary health care providers are able to detect and respond to severe cases of depression during pregnancy. This may improve opportunities to prevent postnatal depression which has significant impact of child outcomes in poorly resourced settings.

The severity of depression seen in this study suggests a likelihood of increased functional loss, warranting consideration of pharmacological, individual or group psychotherapy interventions. Social and educational interventions may be important given that poverty, HIV and intimate partner violence are also common in these settings (Ketchen et al., 2009). However, it is not likely that depression of this severity would respond to social support interventions alone (McKee et al., 2001). Pharmacological interventions are likely important and increasing evidence that complex medical regimes such as HAART can as effectively be delivered and monitored by community health care workers as professionally trained providers (Selke et al., 2010) suggests that similarly creative solutions to the lack of scarce psychiatric skills to deliver pharmacological treatment and monitoring, may be beneficial in these settings.

Further, while individual or group psychotherapy interventions may cost-effectively be placed within centralised primary health care settings, the depression itself may impact of engagement with health care services and hinder compliance or attendance at centrally placed clinic-based mental health services. Task shifting of primary care and prevention functions to community healthcare workers offering decentralised care at a community level can improve the health outcomes of populations at reasonable cost (McPake and Mensah, 2008). This includes successful delivery of treatment for depression by community health workers servicing postnatal women (Rahman et al., 2008a). Harnessing this potential in models of delivery could increase access to treatment while expanding the capacity of health care institutions in resource-constrained environments.

The research is limited by small sample size and the possible selection bias introduced by numbers of women lost to follow up. High rates of depression and the equally high co-morbid HIV may reflect a particular risk profile or factors present in this population of women not generalisable to other antenatal populations with lower perinatal depression or HIV risk.

## Role of funding source

This study was funded by grants from University of Oxford (HQ5035), the Tuixen Foundation (9940), and the Wellcome Trust (082384/Z/07/Z and 071571). These funders had no further role in study design; in the collection, analysis and interpretation of data; in the writing of the report; and in the decision to submit the paper for publication.

## Conflict of Interest

All authors have no conflict of interest to declare.

## Figures and Tables

**Table 1:** Sample characteristics.

Characteristics of participants	N (109)	Percentage
Age		
Median	24	
Range	16–40	
Education		
No education	14	12.8%
Completed primary education	38	34.9%
Some secondary education	32	29.4%
Completed secondary	25	22.9%
Marital status		
Unmarried	100	91.7%
Married	9	8.3%
In stable relationship with partner		
Yes	98	89.9%
No	7	6.4%
Missing	4	3.7%
Cohabiting with father		
Yes	21	19.2%
No	60	55.1%
Missing	28	25.7%
Number of children with father		
First child	57	52.3%
At least one other	52	47.7%
Living arrangements		
Family	87	79.8%
Non family	17	15.6%
Missing	5	4.6%
Regular income		
Yes	54	49.5%
No	55	50.5%
Grant assistance		
Child support grant	52	47.7%
Care dependency grant	1	0.9%
No grant	55	50.5%
Missing	1	0.9%

**Table 2:** Presence of criteria-A and criteria-B symptoms by depression outcome.

Criteria-A symptoms			
**Depressed mood**	Present	**Loss of interest**	Present
Not depressed	19	Not depressed	20
Depressed	49	Depressed	45
Total (%)	68 (62.4%)	Total (%)	65 (59.6%)

*Criteria-B symptoms*			
**Loss of appetite**	Present	**Weight change**	Present
Not depressed	29	Not depressed	54
Depressed	38	Depressed	47
Total (%)	67 (61.5%)	Total (%)	101 (92.7%)
**Energy**	Present	**Sleep disturbance**	Present
Not depressed	27	Not depressed	20
Depressed	42	Depressed	38
Total (%)	69 (63.3%)	Total	58 (53.2%)
**Agitation**	Present	**Retardation**	Present
Not depressed	8	Not depressed	9
Depressed	11	Depressed	21
Total (%)	19 (17.4%)	Total (%)	30 (27.5%)
**Worthlessness**	Present	**Guilt**	Present
Not depressed	12	Not depressed	15
Depressed	31	Depressed	21
Total (%)	43 (39.5%)	Total (%)	36 (33.1%)
**Concentration**	Present	**Decision making**	Present
Not depressed	12	Not depressed	14
Depressed	35	Depressed	28
Total (%)	47 (43.2%)	Total (%)	42 (38.5%)
**Suicide ideation**	Present		
Not depressed	3		
Depressed	27		
Total (%)	30 (27.5%)		

## References

[bb0005] Abiodun O.A. (2006). Postnatal depression in primary care populations in Nigeria. General Hospital Psychiatry.

[bb0010] Alder J., Fink N., Bitzer J., Hosli I., Holzgreve W. (2007). Depression and anxiety during pregnancy: a risk factor for obstetric, fetal and neonatal outcome? A critical review of the literature. The Journal of Maternal-Fetal & Neonatal Medicine.

[bb0015] Almond P., Lathlean J. (2011). Inequity in provision of and access to health visiting postnatal depression services. Journal of Advanced Nursing.

[bb0020] American Psychiatric Association (2000). Diagnostic and Statistical Manual of Mental Disorders (Text Revision).

[bb0025] Austin M.P. (2004). Antenatal screening and early intervention for “perinatal” distress, depression and anxiety: where to from here?. Archives of Women's Mental Health.

[bb0030] Austin M.P., Lumley J. (2003). Antenatal screening for postnatal depression: a systematic review. Acta Psychiatrica Scandinavica.

[bb0035] Barg F.K., Huss-Ashmore R., Wittink M.N., Murray G.F., Bogner H.R., Gallo J.J. (2006). A mixed-methods approach to understanding loneliness and depression in older adults. The Journals of Gerontology Series B: Psychological Sciences and Social Sciences.

[bb0040] Barnighausen T., Tanser F., Gqwede Z., Mbizana C., Herbst K., Newell M.L. (2008). High HIV incidence in a community with high HIV prevalence in rural South Africa: findings from a prospective population-based study. AIDS.

[bb0045] Bennett H.A., Einarson A., Taddio A., Koren G., Einarson T.R. (2004). Prevalence of depression during pregnancy: systematic review. Obstetrics and Gynecology.

[bb0050] Bernazzani O., Conroy S., Marks M.N., Siddle K.A., Guedeney N., Bifulco A., Asten P., Figueiredo B., Gorman L.L., Bellini S., Glatigny-Dallay E., Hayes S., Klier C.M., Kammerer M.H., Henshaw C.A. (2004). Contextual assessment of the maternity experience: development of an instrument for cross-cultural research. British Journal of Psychiatry Supplement.

[bb0055] Bina R. (2008). The impact of cultural factors upon postpartum depression: a literature review. Health Care for Women International.

[bb0060] Brandt R. (2009). Putting mental health on the agenda for HIV + women: a review of evidence from sub-Saharan Africa. Women & Health.

[bb0065] Canino G., Alegría M. (2008). Psychiatric diagnosis — is it universal or relative to culture?. Journal of Child Psychology and Psychiatry.

[bb0070] Carey P.D., Stein D.J., Zungu-Dirwayi N., Seedat S. (2003). Trauma and posttraumatic stress disorder in an urban Xhosa primary care population: prevalence, comorbidity, and service use patterns. Journal of Nervous Mental Disorders.

[bb0075] Cohen L.S., Altshuler L.L., Harlow B.L., Nonacs R., Newport D.J., Viguera A.C., Suri R., Burt V.K., Hendrick V., Reminick A.M., Loughead A., Vitonis A.F., Stowe Z.N. (2006). Relapse of major depression during pregnancy in women who maintain or discontinue antidepressant treatment. Journal of the American Medical Association.

[bb0080] Cook J.A., Grey D., Burke-Miller J., Cohen M.H., Anastos K., Gandhi M., Richardson J., Wilson T., Young M. (2006). Effects of treated and untreated depressive symptoms on highly active antiretroviral therapy use in a US multi-site cohort of HIV-positive women. AIDS Care.

[bb0085] Cooper P.J., Tomlinson M., Swartz L., Landman M., Molteno C., Stein A., McPherson K., Murray L. (2009). Improving quality of mother–infant relationship and infant attachment in socioeconomically deprived community in South Africa: randomised controlled trial. British Medical Journal.

[bb0310] Department of Health, 2010. *National Antenatal Sentinel HIV and Syphilis Prevalence Survey in South Africa, 2009*. Pretoria: National Department of Health. Retrieved on 20th August 2011 from http://www.health-e.org.za/documents/85d3dad6136e8ca9d02cceb7f4a36145.pdf.

[bb0090] Deren S., Oliver-Velez D., Finlinson A., Robles R., Andia J., Colon H.M., Kang S.Y., Shedlin M. (2003). Integrating qualitative and quantitative methods: comparing HIV-related risk behaviors among Puerto Rican drug users in Puerto Rico and New York. Substance Use & Misuse.

[bb0095] Dimatteo M.R., Lepper H.S., Croghan T.W. (2000). Depression is a risk factor for noncompliance with medical treatment: meta-analysis of the effects of anxiety and depression on patient adherence. Archives of Internal Medicine.

[bb0100] Dunkle K.L., Jewkes R.K., Brown H.C., Gray G.E., McIntryre J.A., Harlow S.D. (2004). Gender-based violence, relationship power, and risk of HIV infection in women attending antenatal clinics in South Africa. Lancet.

[bb0105] Esimai O.A., Fatoye F.O., Quiah A.G., Vidal O.E., Momoh R.M. (2008). Antepartum anxiety and depressive symptoms: a study of Nigerian women during the three trimesters of pregnancy. Journal of Obstetrics and Gynaecology.

[bb0110] Evans J., Heron J., Francomb H., Oke S., Golding J. (2001). Cohort study of depressed mood during and after childbirth. British Medical Journal.

[bb0115] Gavin N.I., Gaynes B.N., Lohr K.N., Meltzer-Brody S., Gartlehner G., Swinson T. (2005). Perinatal depression: a systematic review of prevalence and incidence. Obstetrics and Gynecology.

[bb0120] Gotlib I.H., Lewinsohn P.M., Seeley J.R. (1995). Symptoms versus a diagnosis of depression: differences in psychosocial functioning. Journal of Consulting and Clinical Psychology.

[bb0125] Halbreich U., Karkun S. (2006). Cross-cultural and social diversity of prevalence of postpartum depression and depressive symptoms. Journal of Affective Disorders.

[bb0130] Halbreich U., Alarcon R.D., Calil H., Douki S., Gaszner P., Jadresic E., Jasovic-Gasic M., Kadri N., Kerr-Correa F., Patel V., Sarache X., Trivedi J.K. (2007). Culturally-sensitive complaints of depressions and anxieties in women. Journal of Affective Disorders.

[bb0135] Heron J., O'Connor T.G., Evans J., Golding J., Glover V. (2004). The course of anxiety and depression through pregnancy and the postpartum in a community sample. Journal of Affective Disorders.

[bb0325] Houlihan C.F., Bland R.M., Mutevedzi P.C., Lessells R.J., Ndirangu J., Thulare H., Newell M.L. (2011). Cohort Profile: Hlabisa HIV Treatment and Care Programme. International Journal of Epidemiology.

[bb0145] Ickovics J.R., Hamburger M.E., Vlahov D., Schoenbaum E.E., Schuman P., Boland R.J., Moore J. (2001). Mortality, CD4 cell count decline, and depressive symptoms among HIV-seropositive women: longitudinal analysis from the HIV Epidemiology Research Study. Journal of the American Medical Association.

[bb0150] Jewkes R.K., Dunkle K., Nduna M., Shai N. (2010). Intimate partner violence, relationship power inequity, and incidence of HIV infection in young women in South Africa: a cohort study. Lancet.

[bb0155] Kaaya S.F., Mbwambo J.K., Fawzi M.C., van den Borne H., Schaalma H., Leshabari M.T. (2010). Understanding women's experiences of distress during pregnancy in Dar es Salaam, Tanzania. Tanzanian Journal of Health Research.

[bb0160] Kammerer M., Marks M.N., Pinard C., Taylor A., von Castelberg B., Kunzli H., Glover V. (2009). Symptoms associated with the DSM IV diagnosis of depression in pregnancy and post partum. Archives of Womens Mental Health.

[bb0165] Ketchen B., Armistead L., Cook S. (2009). HIV infection, stressful life events, and intimate relationship power: the moderating role of community resources for black South African women. Women & Health.

[bb0170] Kopelman R.C., Moel J., Mertens C., Stuart S., Arndt S., O'Hara M.W. (2008). Barriers to care for antenatal depression. Psychiatric Services.

[bb0175] Kuehn B.M. (2010). Depression guideline highlights choices, care for hard-to-treat or pregnant patients. Journal of the American Medical Association.

[bb0180] Lancaster C.A., Gold K.J., Flynn H.A., Yoo H., Marcus S.M., Davis M.M. (2010). Risk factors for depressive symptoms during pregnancy: a systematic review. American Journal of Obstetrics and Gynecology.

[bb0185] Lau Y., Wong D., Chan K. (2010). The utility of screening for perinatal depression in the second trimester among Chinese: a three-wave prospective longitudinal study. Archives of Women's Mental Health.

[bb0190] Lusskin S.I., Pundiak T.M., Habib S.M. (2007). Perinatal depression: hiding in plain sight. Canadian Journal of Psychiatry.

[bb0195] Madu S., Roos J. (2006). Depression among mothers with preterm infants and their stress-coping strategies. Social Behavior and Personality: An International Journal.

[bb0200] McKee D.M., Cunningham M., Jankowski K.R.B., Zaya L. (2001). Health-related functional status in pregnancy: relationship to depression and social support in a multi-ethnic population. Obstetrics and Gynecology.

[bb0205] McPake B., Mensah K. (2008). Task shifting in health care in resource-poor countries. The Lancet.

[bb0210] Milgrom J., Gemmill A.W., Bilszta J.L., Hayes B., Barnett B., Brooks J., Ericksen J., Ellwood D., Buist A. (2008). Antenatal risk factors for postnatal depression: a large prospective study. Journal of Affective Disorders.

[bb0215] Oates M.R., Cox J.L., Neema S., Asten P., Glangeaud-Freudenthal N., Figueiredo B., Gorman L.L., Hacking S., Hirst E., Kammerer M.H., Klier C.M., Seneviratne G., Smith M., Sutter-Dallay A.L., Valoriani V., Wickberg B., Yoshida K. (2004). Postnatal depression across countries and cultures: a qualitative study. British Journal of Psychiatry Supplement.

[bb0220] Patel V., Abas M., Broadhead J., Todd C., Reeler A. (2001). Depression in developing countries: lessons from Zimbabwe. British Medical Journal.

[bb0225] Patel V., Chisholm D., Kirkwood B.R., Mabey D. (2007). Prioritizing health problems in women in developing countries: comparing the financial burden of reproductive tract infections, anaemia and depressive disorders in a community survey in India. Tropical Medicine & International Health.

[bb0230] Patel V., Simon G., Chowdhary N., Kaaya S., Araya R. (2009). Packages of care for depression in low- and middle-income countries. PLoS Medicine.

[bb0235] Paulden M., Palmer S., Hewitt C., Gilbody S. (2009). Screening for postnatal depression in primary care: cost effectiveness analysis. British Medical Journal.

[bb0240] Posmontier B., Horowitz J.A. (2004). Postpartum practices and depression prevalences: technocentric and ethnokinship cultural perspectives. Journal of Transcultural Nursing.

[bb0245] Prince M. (2008). Measurement validity in cross-cultural comparative research. Epidemiologia e Psichiatria Sociale.

[bb0250] Rahman A., Creed F. (2007). Outcome of prenatal depression and risk factors associated with persistence in the first postnatal year: prospective study from Rawalpindi, Pakistan. Journal of Affective Disorders.

[bb0255] Rahman A., Malik A., Sikander S., Roberts C., Creed F. (2008). Cognitive behaviour therapy-based intervention by community health workers for mothers with depression and their infants in rural Pakistan: a cluster-randomised controlled trial. Lancet.

[bb0260] Rahman A., Patel V., Maselko J., Kirkwood B. (2008). The neglected ‘m’ in MCH programmes — why mental health of mothers is important for child nutrition. Tropical Medicine & International Health.

[bb0320] Rice B.D., Batzing-Feigenbaum J., Hosegood V., Tanser F., Hill C., Barnighausen T., Herbst K., Welz T., Newell M.L. (2007). Population and antenatal-based HIV prevalence estimates in a high contracepting female population in rural South Africa. BMC Public Health.

[bb0265] Rochat T.J., Richter L.M., Doll H.A., Buthelezi N.P., Tomkins A., Stein A. (2006). Depression among pregnant rural South African women undergoing HIV testing. Journal of the American Medical Association.

[bb0270] Sawyer A., Ayers S., Smith H. (2010). Pre- and postnatal psychological wellbeing in Africa: a systematic review. Journal of Affective Disorders.

[bb0275] Selke H.M., Kimaiyo S., Sidle J.E., Vedanthan R., Tierney W.M., Shen C., Denski C.D., Katschke A.R., Wools-Kaloustian K. (2010). Task-shifting of antiretroviral delivery from health care workers to persons living with HIV/AIDS: clinical outcomes of a community-based program in Kenya. Journal of Acquired Immune Deficiency Syndromes.

[bb0280] Smith M.V., Gotman N., Lin H., Yonkers K.A. (2010). Do the PHQ-8 and the PHQ-2 accurately screen for depressive disorders in a sample of pregnant women?. General Hospital Psychiatry.

[bb0285] Starace F., Ammassari A., Trotta M.P., Murri R., De Longis P., Izzo C., Scalzini A., D'arminio Monforte A., Wu A.W., Antinori A. (2002). Depression is a risk factor for suboptimal adherence to highly active antiretroviral therapy. Journal of Acquired Immune Deficiency Syndromes.

[bb0290] Stein A., Krebs G., Richter L., Tomkins A., Rochat T., Bennish M.L. (2005). Babies of a pandemic. Archives of Diseases of Childhood.

[bb0295] Tanser F., Hosegood V., Barnighausen T., Herbst K., Nyirenda M., Muhwava W., Newell C., Viljoen J., Mutevedzi T., Newell M.L. (2008). Cohort profile: Africa Centre Demographic Information System (ACDIS) and population-based HIV survey. International Journal of Epidemiology.

[bb0300] Vesga-Lopez O., Blanco C., Keyes K., Olfson M., Grant B.F., Hasin D.S. (2008). Psychiatric disorders in pregnant and postpartum women in the United States. Archives of General Psychiatry.

[bb0305] World Health Organisation (2009). Mental health aspects of women's reproductive health: a global review of literature.

